# The role of bodily self-consciousness in episodic memory of naturalistic events: an immersive virtual reality study

**DOI:** 10.1038/s41598-023-43823-2

**Published:** 2023-10-09

**Authors:** Sylvain Penaud, Delphine Yeh, Alexandre Gaston-Bellegarde, Pascale Piolino

**Affiliations:** Université Paris Cité, Laboratoire Mémoire, Cerveau & Cognition (LMC2 UR 7536), Institut de Psychologie, 71 Ave Édouard Vaillant, 92100 Boulogne-Billancourt, France

**Keywords:** Psychology, Human behaviour

## Abstract

Recent studies suggest that the human body plays a critical role in episodic memory. Still, the precise relationship between bodily self-consciousness (BSC) and memory formation of specific events, especially in real-life contexts, remains a topic of ongoing research. The present study investigated the relationship between BSC and episodic memory (EM) using immersive virtual reality (VR) technology. Participants were immersed in an urban environment with naturalistic events, while their visuomotor feedback was manipulated in three within-subjects conditions: Synchronous, Asynchronous, and No-body. Our results show that asynchronous visuomotor feedback and not seeing one’s body, compared to synchronous feedback, decrease the sense of self-identification, self-location and agency, and sense of presence. Moreover, navigating in the Asynchronous condition had a detrimental impact on incidental event memory, perceptual details, contextual association, subjective sense of remembering, and memory consolidation. In contrast, participants in the No-Body condition were only impaired in egocentric spatial memory and the sense of remembering at ten-day delay. We discuss these findings in relation to the role of bodily self-representation in space during event memory encoding. This study sheds light on the complex interplay between BSC, sense of presence, and episodic memory processes, and strengthens the potential of embodiment and VR technology in studying and enhancing human cognition.

## Introduction

Like every workday, you are sitting in a cafe enjoying your coffee when suddenly the memory of a brief conversation that took place in the same cafe comes to mind. In addition to recalling objective details about the conversation, yourself, and the other people involved, as well as contextual information about the specific place and time the event occurred, this meaningful representation also includes subjective vivid perceptual details, emotional states, and thoughts, all bound together in a rich multidimensional reexperience^1–4^. Hence, your memory comes with a sense of self. Indeed, when we remember a personal event from our past, we not only undergo the experience of being the protagonist of a conscious episode but also of being the subject in the memory^5^. According to Tulving, this subjective experience linked to the memory of everyday events that occurred at particular times and places is a hallmark of episodic memory (EM), which is closely related to what researchers refer to as episodic autobiographical memory^6^. It is supported by autonoetic consciousness (i.e., the ability to remember previous events as a sense of self-recollection), a specific form of self-consciousness that allows one to mentally travel in time and reexperience specific events from one’s past. Moreover, autonoetic consciousness distinguishes EM from semantic memory, which is supported by noetic consciousness (i.e., the ability to know facts without recollection)^7^.

The central role of the self in EM retrieval raises questions about the influence of self-consciousness during the process of initial encoding^8,9^. Numerous studies have explored the importance of cognitive self-referencing in the process of encoding and retrieving episodic memories, which is commonly referred to as the self-reference effect^10,11^. Over the years, self-reference has been shown to enhance the recall of objective and subjective information^12^, and to promote associative memory (i.e., feature binding)^13^. However, other dimensions of selfhood have been overlooked. Indeed, it has been emphasized that experiences assume an “I” who is the subject of the ongoing flow of events^14,15^. This minimal sense of self is characterized by the pre-reflective feeling of being a self in a body, dissociated from its environment and providing a sense of presence in the world^16,17^. Over recent decades, convergent findings from psychology and neuroscience have demonstrated that this minimal sense of self is flexible, plastic, and depends upon multi-sensory integration processes that combine bodily signals^18–20^. Research has consistently demonstrated that simultaneous visual and tactile or motor stimulation that is both spatially and temporally congruent can trigger body illusions defined as an incorporation of an extra-corporeal body part or even an entire body into the sense of bodily-self^21,22^. Central features of body self-consciousness (BSC) encompass the sense of identification with one’s own body (i.e., *self-identification*), the sense of being located in space and time (i.e., *self-location*), the feeling of having control over one’s own body (i.e., *agency*) and the centeredness of experience around the body (i.e., *first-person perspective*)^23^.

Proponents of the embodied approach of EM argue that bodily and sensorimotor information is integrated into the memory trace and later reactivated to support memory retrieval^24–26^. Several recent theoretical frameworks on memory and the self have also stressed the influence of bodily processes and BSC on autonoetic consciousness and mental time travel^27–32^. For example, Prebble, Addis and Tippett argued that episodic recollection “requires the sensory-perceptual and internal aspects of the original experience to be encoded from a subjective, egocentric perspective”^27^. Thus, according to these authors, BSC is a necessary precursor of higher order and extended forms of selfhood. In addition, a growing amount of clinical evidence indicates that compromised BSC is associated with impairments in EM, as observed in individuals with schizophrenia or depersonalization disorders^27,33^.

Noteworthy, the rapid growth and broad accessibility of computer-based 3D technologies have enabled researchers to make significant progress in understanding memory and the self. Devices such as Virtual Reality (VR) provide many advantages for evaluating EM^34,35^. VR is a computer-generated immersive technology that enables users to interact with a simulated world in a three-dimensional space, providing a sense of presence or “being there”^36,37^. It allows researchers to create self-involving experiences and simulate expansive and ecologically valid environments that better capture how cognition operates in real-world settings over various populations, including children, young adults, and healthy and pathological aging^38–42^. Moreover, VR allows for the presentation of complex stimuli such as 3D objects and everyday-like scenes that provide the opportunity for evaluating the multidimensional and associative nature of EM in naturalistic settings while maintaining a high degree of experimental control^43^. Accordingly, it has been shown that assessments of EM using VR were more reliably associated with daily memory complaints in healthy aging and pathological populations and were more sensitive to memory impairments than standard neuropsychological tools. Furthermore, knowledge acquired in VR is more easily transferable to the real world^44^. VR has also established itself as an embodied technology, allowing us to assess the influence of the body on cognition^45^. For example, the enactment effect—the finding that actively navigating in a virtual environment enhances EM compared to passively observing—generally improves factual, contextual, and associative memory and the sense of remembering^46,47^. In more recent times, scientific research has used the affordances of VR technology to manipulate the synchrony of bodily signals, producing a complete bodily illusion over a virtual avatar perceived from a first-person perspective (1PP). This form of bodily illusion has been employed to investigate the role of body self-consciousness in EM^48–53^ (see Table 1).

**Table 1:** Summary of the methodology and main findings of previous studies.

Studies	Stimuli	Encoding	Method	Main findings
Tuena et al.^48^	Virtual scenes	Incidental	High vs. Medium vs. Low embodiment	High > Medium and Low embodiment for source and hit rate (immediate)
Bréchet et al.^49,50^	3D objects	Incidental	Body vs. No-Body	Body > No-body in a delayed recognition task
Gauthier et al.^51^	3D objects	Incidental	Body vs. No-Body	Body = No-body in a recognition task (immediate and delayed)
Tacikowski et al.^52^	Trait-adjectives	Incidental	Synch vs Asynch V-T stimulation	Synch > Asynch in a recognition task (immediate)
Iriye and Ehrsson^53^	Video scenes	Intentional	Synch vs Asynch V-T stimulation	Synch > Asynch for details, reliving (immediate), vividness, emotional intensity, and confidence (one week delay)

Synch, synchronous; Asynch, asynchronous; V-T, visuo-tactile.

For example, Bréchet and colleagues used a head-mounted display to immerse participants in a sequence of virtual rooms in which a collection of everyday-life objects were organized. During the encoding phase of the experiment, participants were instructed to explore each room freely. Half of the subjects experienced the integration of their physical body in the virtual environment from a first-person perspective, while the other half did not have any visible body representation. Then, participants were immersed again in the same rooms for a retrieval session in which they underwent a surprise forced-choice recognition task immediately or one hour after the encoding session. The results indicated that participants recalled fewer objects when the participants' physical bodies were not visible during encoding, but only with a 1-h delay^49^. Additionally, in a subsequent neuroimaging investigation, the same group demonstrated that observing one’s own body during the process of encoding was linked to a higher connectivity between the brain regions associated with BSC and EM^51^. The authors conclude that the multisensory stimulations associated with the perception of one’s body in 1PP are essential for memory encoding.

More recently, Tacikowski et al. (2020)^52^ asked pairs of friends to complete a personality-trait judgment task serving as an incidental encoding. The HMD device was connected to two sets of cameras so that participants could see either their own or the friend’s body in a supine position from a first-person perspective. Depending on the experimental condition, the seen body was stroked with a wooden stick either synchronously or asynchronously to manipulate sense of body ownership. Next, participants were asked to complete a body-ownership questionnaire and underwent an unattended recognition task. Their results showed that participants who received synchronous stroking during the incidental learning phase performed better in the recognition task. Moreover, changes in memory performances during the recognition task were positively correlated with rating changes in body ownership between the synchronous and asynchronous conditions^52^.

Lastly, Iriye and Ehrsson^53^ presented a series of videos using a head-mounted display that depicted realistic everyday life scenarios. Each scenario consisted of a stereoscopic view of a mannequin’s body seen from a supine position and aligned with the participant’s actual body. During the scenarios, participants observed a wooden stick repeatedly stroking the mannequin at a regular pace, either synchronously or asynchronously. Subsequently, participants were administered a cued recall test immediately and after one week to assess memory accuracy for both central and peripheral details. Participants also rated their memories on seven-point Likert scales for reliving, emotional intensity, vividness, and confidence in memory accuracy. The authors found that synchronous stroking improved memory accuracy regardless of the time delay between the encoding and retrieval of information. Additionally, analysis of subjective measures indicated that participants remembered the encoded event with a stronger sense of reliving immediately after the encoding session and with higher emotional intensity and confidence after a one-week delay. Taken together, these findings demonstrate that the sense of owning a virtual body observed from a first-person perspective during encoding has an impact on the encoding of both objective and subjective elements of EM. Nevertheless, various issues remain to be addressed.

First, the studies mentioned above mostly used simple stimuli such as words or 3D objects or intentional encoding instructions that do not account for EM complexity and multidimensionality in everyday life, thereby deviating from its functioning in real-world situations. To date, only one study has used full embodiment and incidental encoding of virtual scenes^48^. This study from our team compared the impact of high, medium, and low levels of embodiment on source memory and hit rate. The results indicated that participants in the high embodiment condition trended towards superior performance compared to those in the medium and low embodiment conditions. However, the sense of embodiment was not measured in all conditions, which makes it challenging to link memory performance to bodily self-consciousness. Additionally, the study’s sample size was small, and the materials used (Kinect) did not allow for perfect real-time tracking of bodily movements. Second, the studies above used simplistic encoding material, which does not enable the complete examination of the associative component of EM^54,55^. The integration of spatial and temporal information encoding, along with associative mechanisms that bind items and contextual information, is at the core of EM^56^. Interestingly, recent studies have shown that BSC may influence both spatial^57^ and temporal processing^58^, as well as their association in the hippocampus^59^. Therefore, assessing contextual information is crucial to understand better how BSC affects EM. Third, the studies mentioned above focused exclusively on the impact of body ownership. However, other studies on BSC and EM have demonstrated that spatial, including sense of self-location^59^ and perspective^9^, and agentive components of BSC may also play a role in EM encoding. Moreover, changes in self-location may occur even when participants are embodied in first-person^60^. Hence, a comprehensive evaluation that encompasses all aspects of somatic self-awareness is crucial for gaining a better comprehension of its function in EM and its distinct constituents. Lastly, the literature reported mixed findings regarding the effect of delay and used different types of memory tests and experimental designs, which makes it difficult to compare them.

In this experiment, we sought to explore the role of BSC on EM in conditions close to its expression in daily life, examining incidental memory of new events experienced in a naturalistic controlled environment. We were especially interested in assessing complex EM traces by considering the objective and subjective components (event details, contextual information, and sense of reexperiencing). We used HMD to embody participants in a personalized avatar seen from a first-person perspective and manipulated visuo-motor feedback to modulate BSC. Visuomotor stimulation has been widely used to induce body-part and full-body illusions^61,62^. Like visuo-tactile stimulation, visuomotor stimulation relies on the spatiotemporal congruence between the seen and the felt movements. However, in contrast to visuo-tactile stimulation, visuomotor stimulation conveys a variety of bodily signals that goes beyond the integration of mere visual and tactile information. It provides information on how the relative position and orientation of the real and fake bodies change over time, allowing individuals to track how the body moves in relation to the environment. Moreover, visuomotor signals bear significant signals for the self-other distinction and have been shown to produce stronger and more stable changes in BSC (for a review, see Kilteni et al.^63^). Finally, visuomotor stimulation is more ecologically valid than visuo-tactile stimulation, as it more closely resembles the way in which we typically experience the world.

Thus, using a within-subjects design, participants were immersed in a personalized avatar’s body, either synchronized, desynchronized, or occluded from the participant’s view during their walk in the virtual environment. In light of the results mentioned above, we hypothesized first that participants in the Asynchronous and No-Body conditions would report lower self-identification, self-localization, agency, and sense of presence than those in the Synchronous condition. Second, for the memory tests, we hypothesized that participants would recall fewer events and less contextual information and report a weaker sense of remembering and associated phenomenology in the Asynchronous and No-Body conditions compared to the Synchronous condition. Finally, as our study is the first to contrast Asynchronous and No-Body conditions directly, there is no clear evidence to anticipate there would be any differences between the two conditions for any of our measures. Similarly, we did not have expectations regarding the interaction between BSC and recall’s delay.

## Method

### Participants

A total of 38 young adults (11 males; mean age: 22.11 yo ± 4.77) were recruited for this experiment. Two participants were removed from the pool due to cybersickness issues, and two others as they did not attend the second session. Thus, the final number of participants was 34 (10 males; m: 22.11 yo (4.75)). All participants had normal or corrected visual acuity, declared no history of neurological and psychopathological disorders, and were blind to the purpose of the experiment. Participants were recruited from the University of Paris-Cité and from the Relais d’Information sur les Sciences de la Cognition (RISC). All participants signed informed consent and received course credits or a 20€ voucher in exchange for their participation. All the experimental procedures and methods were approved by the Ethical Research Committee of Paris-Cité University (N° IRB: 0012021-107) and have been conducted in accordance with the Declaration of Helsinki.

### Material

**Figure 1:**
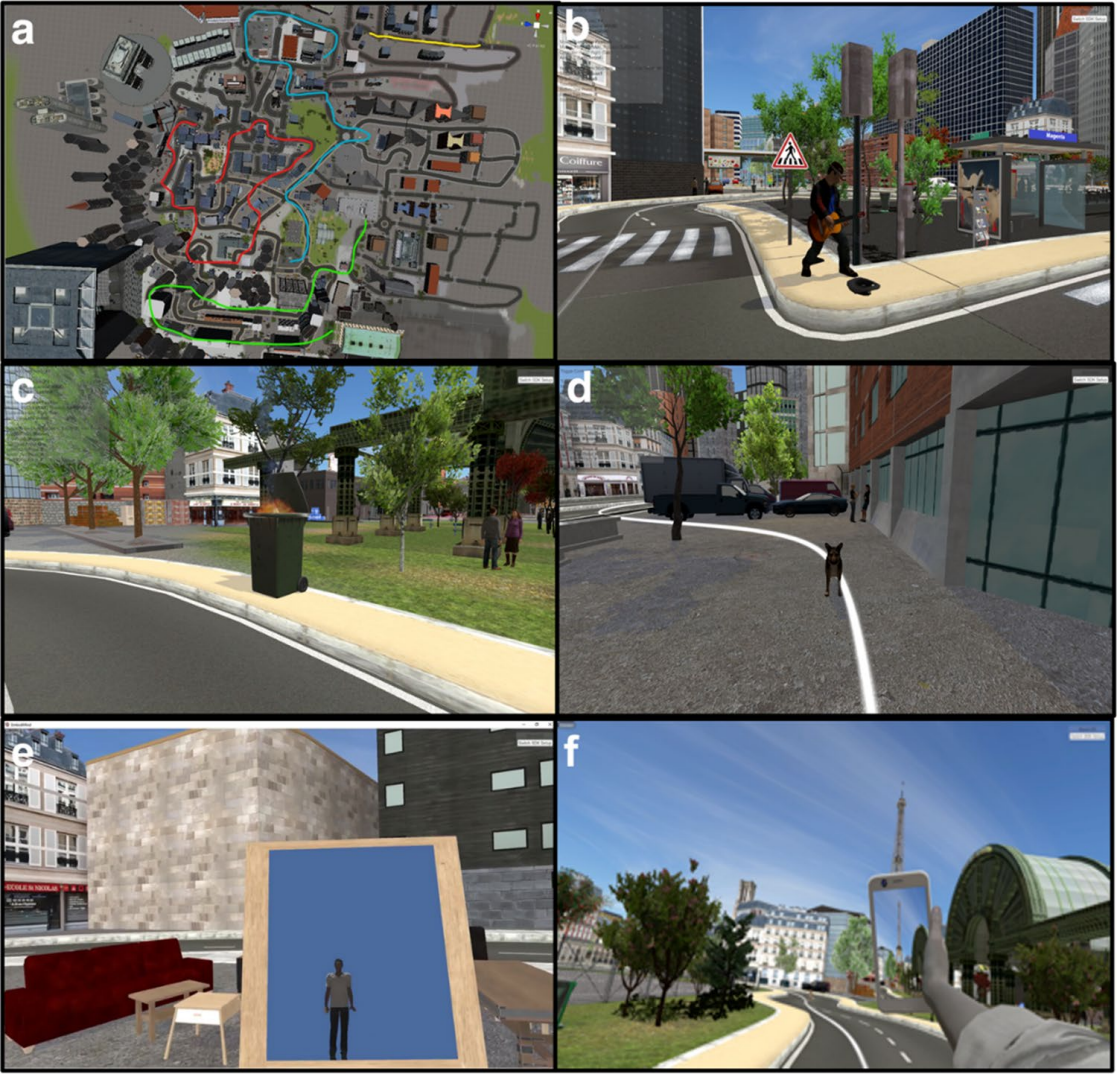
Study paths and events—(**a**) Map of the three different paths used in the study (red, blue, and green; the yellow path is for training). (**b**) Virtual event of a man playing the guitar. (**c**) Virtual event of a trashcan on fire. (**d**) Virtual event of a dog barking aggressively. (**e**) First-person view of the mirror. Participants are asked to stop in front of each mirror and raise each of their limbs once. (**f**) First-person view of the cellphone. Images and map were obtained in the MC2 Lab using UNITY v2019.2.5f1 software.

**Figure 2:**
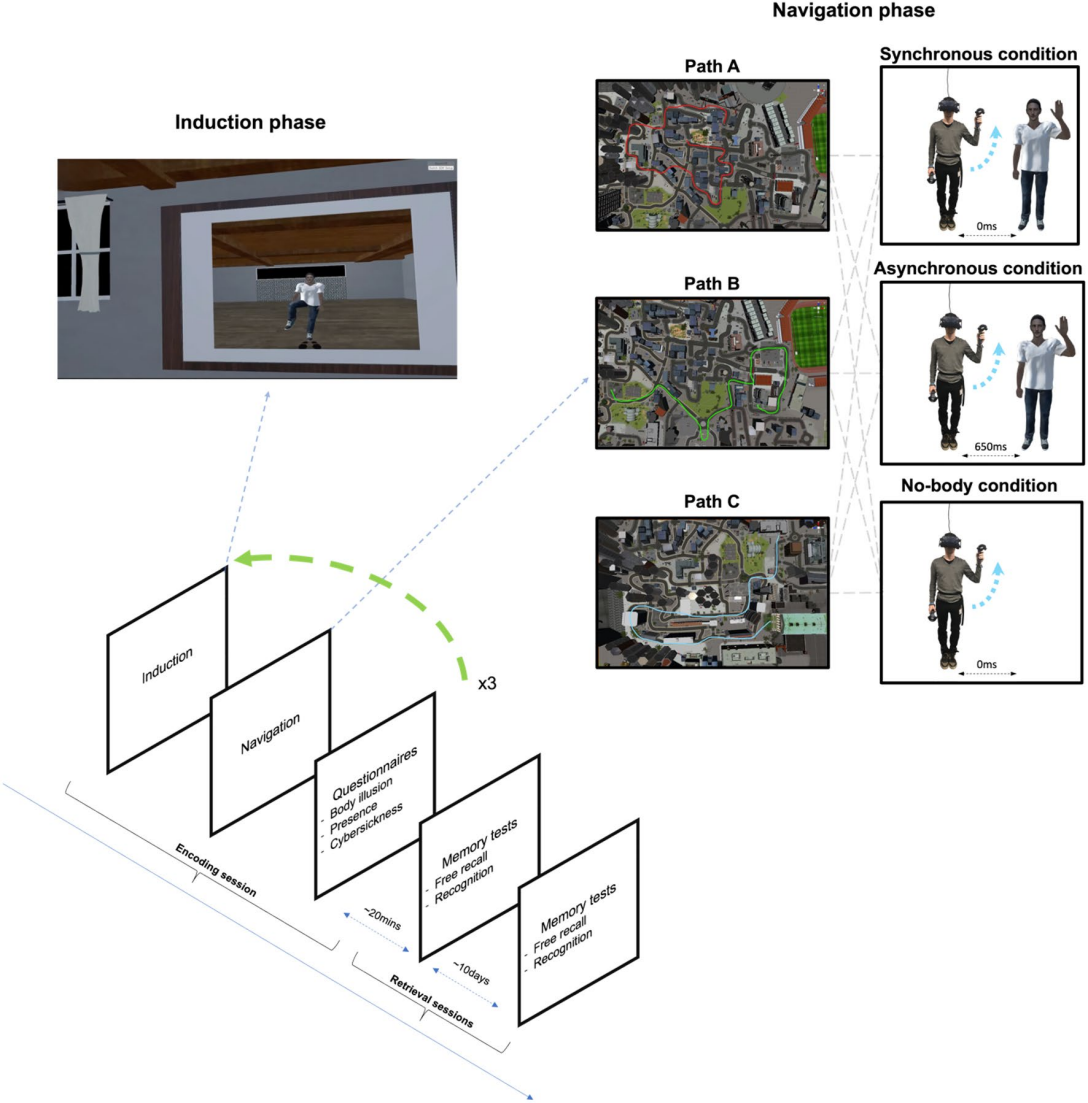
Experimental design—the experiment has a within-participant design. All sessions started with an induction phase in front of a virtual mirror. In the No-Body condition, there was no reflection of the participant in the mirror (see induction phase). Next, participants were assigned to one of the paths in a counterbalanced order. After each navigation, participants completed the body-illusion, presence, and cybersickness questionnaires. This procedure was repeated for each experimental condition. Once all the navigation tasks had been completed, participants were administered an unattended free recall and recognition task, both immediately and 10 days after the encoding session. Figure and maps designed in the MC2 Lab using UNITY v2019.2.5f1 software.

The virtual environment was built in our lab using Unity 2019.2.5f1 software. The environment was designed to immerse participants in a close-to-daily-life urban landscape with rich ambient sounds, animations, and scenes displayed on an HTC Vive Pro headset equipped with headphones (stereoscopic). We further circumscribed three paths of approximately equal length (~ 5 min) at different locations in the virtual environment (Fig. 1a). A bright white line was visible on the ground to delineate all paths and indicate the route to follow. Each path included 12 short audiovisual animated scenes representing everyday-life events for a total of 36 scenes (Fig. 1b–d). The scenes depicted characters, animals, or objects engaged in daily-life situations (e.g., a street musician playing the guitar, a trashcan on fire, or a group of friends saying “Hello”) and were evenly distributed around the white line so that as many events were on the left, right, or in front of the participants. Animations started when participants entered a defined area around the events and were played only once. Three mirrors were also placed respectively at one-third, halfway, and two-thirds along each path. The purpose of these mirrors was to maintain multi-sensory stimulation as body illusion may decay over time^64^ (see Procedure below and Fig. 2a). Virtual avatars were created using Character Creator 3. Each avatar was personalized by means of a headshot sent by the participants a few days before the experiment. This was meant to increase participants’ identification with virtual avatars. The photo was matched (gender, age, skin color, haircut, hair color, and facial traits) on the avatar’s body using the Character Creator 3 headshot functionality. Participants’ body movements were tracked in real-time by means of five HTC-Vive sensors installed on participants’ heads, hands, ankles, and waists, and two base stations installed at the corners of the experimental room.

#### Behavioral data acquisition

Free recalls were registered using audio recording software for later scoring. The recognition tasks were implemented using Neuropsydia software^65^ and presented on a 15.6″ laptop screen.

#### Procedure

The experiment comprised two sessions approximately ten days apart (m = 9.34; sd = 3.7; min = 4, max = 21) (Fig. 2).

#### Encoding session

Upon arrival, participants signed an informed consent form and were given the task instructions. Participants were told that they would be immersed successively in three different parts of a city and that they should visit each as if they were planning to live there (incidental encoding). They were asked to pay careful attention to each path and the events that might occur in it as we would question them on which district they preferred and why at the end of the experiment. Next, participants were fully equipped with VR sensors, controllers, and headset.

### Training

Before starting the experimental navigation, participants were first immersed in a training environment to familiarize them with the virtual city and the controls. The training session took place in a dedicated path similar to, but not overlapping with, the path used in the experimental condition. The training comprised three events not used for the experimental conditions and one mirror. Mirrors were marked with a flashing panel, and participants were asked to stop in front of each mirror to raise each of their limbs once, first looking at their reflection in the mirror, then looking directly at their limbs to actualize embodiment (Fig. 1e). Moreover, a virtual cellphone was added in the right hand of the virtual avatar during the training session and experimental navigations (Fig. 1f). Participants were told they could freely use the cellphone to take pictures in the environment by pressing a trigger placed behind the right controller. The purpose of the cellphone was to encourage participants to use their virtual bodies to maintain multi-sensory stimulation throughout the navigation.

#### Experimental session

Once the participants had completed the training, they were immersed in a virtual empty room equipped with a large virtual mirror from a first-person perspective. Each participant successively underwent each of three experimental conditions in a counterbalanced order (see Induction phase and Fig. 2). In the Synchronous Avatar condition, the movements of the virtual avatar were visible and fully synchronized with the participant’s head, arms, legs, and waist movement. In the Asynchronous Avatar condition, the virtual avatar was visible, but a 650 ms delay was introduced between the participant and the avatar’s movements. This delay was chosen because a pilot study revealed that shorter asynchrony (i.e., 500 ms) failed to differentiate synchronous from asynchronous ratings on the body-illusion questionnaire. Finally, in the No Avatar condition, the virtual avatar’s body was not visible from the participant’s view. Importantly, in the Asynchronous condition, a 650-ms delay was also introduced between the participant’s movements and the cellphone, as well as between the trigger push and the camera’s click sound. In the No-Body condition, the cellphone was fully synchronized with the participant’s movements; thus, it was visible at the location of the (not visible) participant’s avatar hand.

### Induction phase

The induction phase was designed to induce the body illusion over the virtual avatar depending on the experimental condition before experimental navigation started. To this end, participants were immersed in a room equipped with a large mirror. The induction started with a calibration procedure to ensure the correct positioning of the avatar relative to the participant’s body. Next, participants were instructed to slowly raise each of their arms and legs five times in front of the mirror, once looking at their reflection in the mirror and then looking directly at the avatar’s body. For the No-Body condition, participants were asked to raise each of their limbs five times *as if* they were seeing them in the mirror and then *as if* they were seeing them directly. Thus, the induction phase was identical in all conditions except for body visibility and synchrony.

#### Navigation

After completing the induction phase, participants were immersed in the assigned virtual path. Every navigation started with a new calibration. After each navigation, the participant completed three short questionnaires assessing body illusion, sense of presence, and level of cybersickness.

### Questionnaires

The body-illusion questionnaire included five statements and was adapted from various existing questionnaires to accommodate ratings on visible and invisible virtual bodies^53,66^ (see Table 2). Q1 (“I felt I had control over the body under the virtual reality headset/my head”), Q2 (“I felt that the body under the virtual reality headset/my head was my body”), and Q3 (“I had the feeling that I was located in the same place as the body under the virtual reality headset/my head”) assessed the sense of self-identification with the avatar, sense of agency, and sense of self-location, respectively. C1 (“I had the feeling that I couldn’t feel my body anymore”), and C2 (“I felt as if I had two bodies”) were control statements assessing compliance and suggestibility. Participants were asked to rate each statement on a 7-point Likert scale (− 3 = Strongly Disagree, 0 = Neutral, 3 = Strongly Agree). Sense of presence was measured using a short version of the Igroup Presence Questionnaire (IPQ)^67^, and cybersickness using the French version of the Simulator Sickness Questionnaire^68^.

**Table 2:** Body-illusion questionnaire—Q1, Q2, and Q3 assess self-identification, self-location, and sense of agency respectively.

Body illusion questionnaire	
Sometimes during navigation…	
Q1	I felt I had control over the body under the virtual reality headset/my head (Agency)
Q2	I felt that the body under the virtual reality headset/my head was my body (Self-identification)
Q3	I had the feeling that I was located in the same place as the body under the virtual reality headset/my head (Self-location)
C1	I had the feeling that I couldn’t feel my body anymore (Control)
C2	I felt as if I had two bodies (Control)

C1 and C2 are control questions.

#### Retrieval session

Once all three navigations were completed, participants underwent an unattended free recall and recognition test. The free recall test lasted 20 min and was adapted from a Virtual Reality EM questionnaire previously validated by our team^9^. Participants were asked to recall as many events as possible in the allotted time while being recorded for later scoring. To assess contextual egocentric spatial and temporal information, participants were further asked to indicate if the events took place on their right, on their left, or in front of them (egocentric spatial situation) and had to roughly tell if the event happened at the beginning, in the middle, or at the end of a given district (temporal situation). Finally, they were asked to provide as many specific details as possible about each event. Details could be visual or auditive perceptual details, characters’ actions, or details pertaining to the background of the scene.

For the recognition task, the 36 old events were presented in a random order mixed up with 18 new events for a total of 54 trials. This ratio has been chosen because it accommodates better The new events were like the old events regarding the types of content and complexity and could have been used interchangeably with the events used in the navigations (e.g., A man playing football, two peoples laughing out loud in the street). The stimuli were presented on the center of a white 15.6″ laptop screen. Background information was removed from the events to avoid contextual cues. For each event, participants had to indicate if it was seen before in one of the three paths by clicking on the right mouse button. When the event was correctly recognized, participants were further asked to indicate the source (condition of encoding) and their degree of remembering (i.e., reexperiencing) on a continuous scale (0 = I don’t remember, I know; 100 = I fully remember)^69^, which is a more fine-grained measure of remembering experienced events than the classic dichotomous Remember/Know paradigm^7^ used for simple material such as words^70^. Finally, each event with a remember score > 50 was presented again in random order. To assess egocentric spatial memory, the participants were asked to indicate if the event happened on their left, right, or in front of them. For temporal memory, three events pertaining to the same path were presented on the upper part of the screen and numbered randomly from 1 to 3. The participants had to choose the correct order of appearance of the events among all six possible orders noted in the lower part of the screen (e.g., 2–3–1). We only asked for contextual information on the correct recognized events to avoid participants deducing old and new events based on the temporal order task: because all the events presented in a single trial were part of the same path, participants could have deduced the old event based on this information. For source memory, participants were asked to indicate whether the event pertained to the first, second or third navigation. Lastly, participants assessed each past event they had previously recognized and associated to a Remembering score greater than 50 with respect to first-person perspective, vividness, confidence in memory, emotional intensity and memory ownership using a series of Likert 0–100 scales.

Finally, the participants completed an unattended free recall and recognition test in the same way approximately ten days later.

### Scoring

For the scoring of the free recall and the recognition tasks, all the measures were computed for each of three conditions (Synchronous, Asynchronous and No-Body): one point was attributed for every correctly recognized or recalled event (what), spatial egocentric (where), and temporal information (when). Specific details for each event were scored up to a maximum of three points (one point per detail). This maximum was decided based on a pilot study. Importantly, details had to be specific to the remembered event (e.g., reporting that characters in a scene representing a meditating group were in a lotus position was not considered specific as it is common to most meditating situations, whereas saying that one character was wearing a white and green shirt was considered specific). Each score was then normalized by dividing it by the maximum attainable score and was expressed as a percentage to account for individual performances. For example, the “What” score of a participant was determined by dividing the count of correctly retrieved events by the highest attainable score (i.e., 12). In contrast, the scores for “Where,” “When”, “Details” and “Source” were computed by dividing the number of correct responses for each of these categories by the number of “What” responses for that particular condition. For the recognition task, we further calculated a d-prime score by subtracting the Z score of the false-alarm rate from the Z score of the hit rate to assess overall performance.

To assess the richness of contextual information, we calculated a total associative memory score that correspond to the mean amount of information category per condition in the free recall task : 1 point was given for each information category associated with What (i.e., where, when, and at least one specific detail) and summed up for each event out of a maximum of 3 points. For example, a participant recalling an event with its spatial egocentric location and in the correct temporal order would have an associative memory score of 2 points. If the participant further recalled at least one specific detail, this raised the associative memory score to 3 points. We then calculated the mean amount of information per condition and expressed this score as a proportion of the maximum possible given the “What” responses.

### Statistical analysis

We performed a mixed model analysis that included the within factors of Condition (Synch, Asynch, and No-Body). We also examined the interaction between Condition and Delay (Immediate or Delayed) for the memory tests by adding Delay as an additional within factor. Mixed models are well suited for repeated measure designs as they enable dependencies in the data to be considered. Models were computed using the *lmer* function from the *lme4* package in R studio version 4.0.4. When data residuals were not normally distributed (as assessed by a Shapiro–Wilk test), we used a robust version of the *lmer* function named *rlmer* using the *robustlmm* package^71^. Estimates, confidence intervals, and p-values for fixed effects estimates were computed using a Wald t-distribution approximation using the parameters function of the performance package^72^. Marginal and conditional R^2^ were computed following the method prescribed by Nakagawa and Schielzeth^73^. Pairwise comparisons and adjusted means were obtained with the *emmeans* package, version 1.7.5^74^. The alpha level was set at 0.05, and p-values were adjusted for multiple comparisons using the Holm-Bonferroni correction. The “Results” section presents significant results only. Non-significant results are accessible in the supplementary material (see Supplementary Material).

## Results

### Control check for conditions

We first checked for potential differences in cybersickness and navigation between conditions. To assess differences in cybersickness, we fitted a hierarchical mixed model on the main and the sub-scores of the Simulation Sickness Questionnaire (SSQ) with Condition as a fixed effect and subjects as a random intercept. Importantly, none of our subjects exceeded the SSQ threshold for cybersickness. Our model revealed no significant predictor of condition on the oculomotor sub-type of the SSQ. For the nausea sub-type, our model indicated that the Asynchronous (β = 0.62, SE = 0.25, CI(95%) = [0.14, 1.10], t = 2.52, p = 0.012) and No-Body conditions (β = 0.58, SE = 0.25, CI(95%) = [0.10, 1.06], t = 2.37, p = 0.018) were significant positive predictors of nausea score (η2c = 0.83, η2m = 0.007). Pairwise comparisons showed that participants in the Asynchronous condition had a higher feeling of nausea compared to those in the Synchronous condition (M_asynch_ = 3.84, SE = 0.564, M_synch_ = 3.22, SE = 0.564, β = − 0.6172, SE = 0.245, p = 0.0356) and No-Body condition (M_nob_ = − 3.80, SE = 0.564, β = − 0.5807, SE = 0.245, p = 0.0358). For the total cybersickness score, our model indicated that the Asynchronous (β = 0.95, SE = 0.46, CI(95%) = [0.06, 1.85], t = 2.08, p = 0.037) and the No-Body conditions (β = 0.91, SE = 0.46, CI(95%) = [0.02, 1.81], t = 2.00, p = 0.046) were significant positive predictors of total cybersickness score (η2c = 0.83, η2m = 0.005). However, pairwise comparisons did not indicate any significant differences between conditions (all p > 0.05). Regarding the oculomotor subtype, neither being in the Asynchronous (β = − 1.12, SE = 2.71, CI(95%) = [− 6.46, 4.23], t = − 0.41, p = 0.681) nor in the No-Body condition (β = -0.56, SE = 2.66, CI(95%) = [− 5.81, 4.70], t = − 0.21, p = 0.835) were significant predictors of oculomotor cybersickness.

To assess differences in navigation duration between conditions, we fitted the same model with navigation duration as the dependent variable and SSQ nausea as a covariate to control for differences in cybersickness. Analysis of navigation duration showed that being in the Asynchronous condition was a positive predictor of navigation duration (β = 0.79, SE = 0.30, CI(95%) = [0.53, 1.05], t = 5.99, p < 0.001; η2c = 0.81, η2m = 0.085). Pairwise comparisons showed that navigation duration was higher in Asynchronous compared to the Synchronous (M_synch_ = 8.44, SE = 0. 273, M_asynch_ = 9.23, SE = 0. 273; β = − 0. 7941, SE = 0.133, p < 0.0001) and the No-Body condition (M_nob_ = 8.44, SE = 0. 273; β = 0. 8385, SE = 0.131, p < 0.0001). Therefore, we decided to add the navigation duration and SSQ Nausea sub-scores as two covariates in all the subsequent models.

### Body illusion

**Figure 3:**
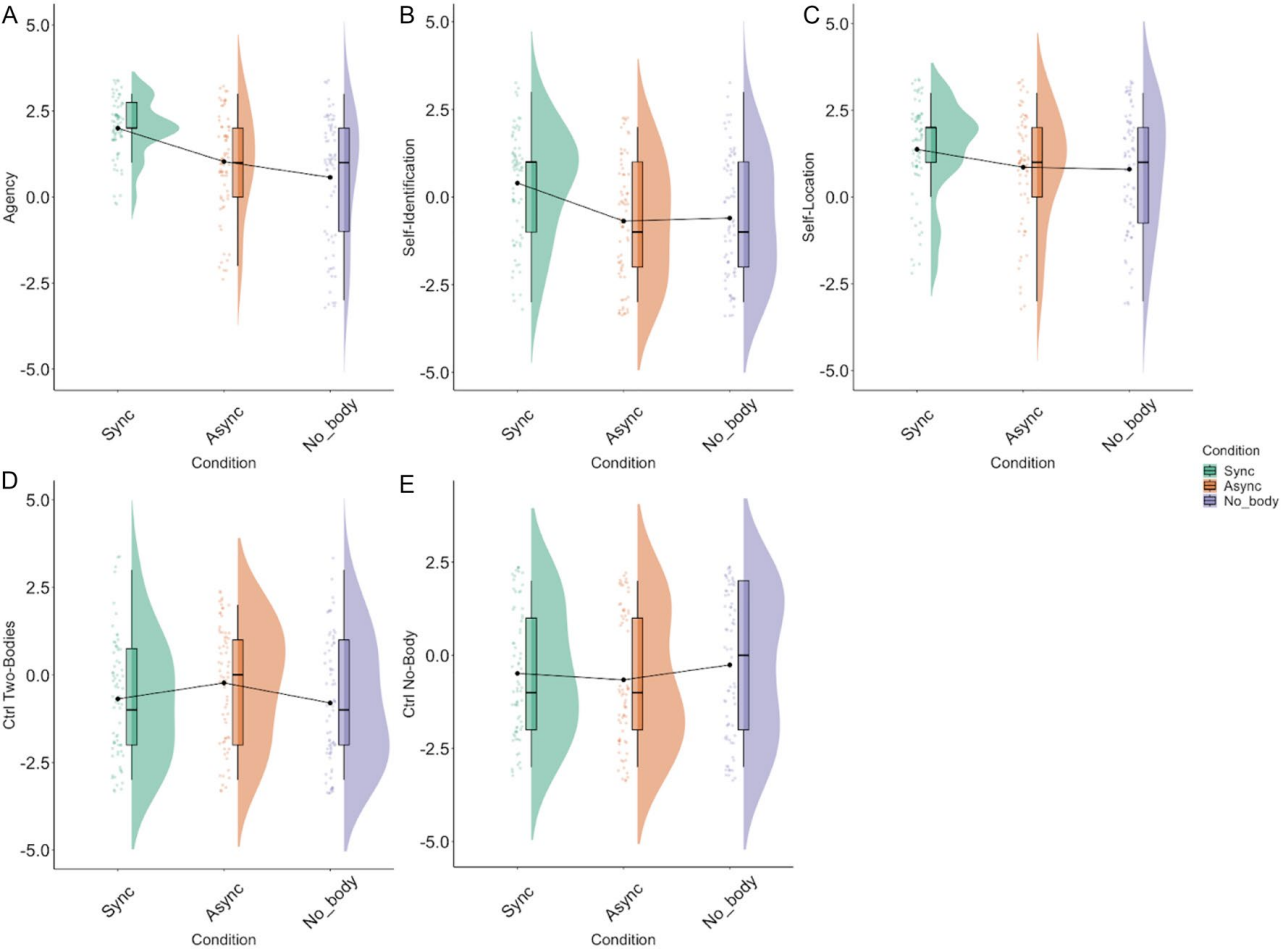
Results of the Body illusion questionnaire.

First, to ensure that our experimental manipulation worked as expected, we conducted an analysis of the body-illusion questionnaire responses after each navigation. To analyze the body-illusions score, we fitted a model with Condition as a fixed effect and Subjects as a random intercept. Based on our previous results, we also included Navigation Duration and SSQ Nausea sub-scores as covariates in the model. Analysis of sense of body self-identification showed that being in the Asynchronous (β = − 1.09, SE = 0.23, CI(95%) = [− 1.54, -0.64], t(203) =  − 4.74, p < 0.001) and No-Body conditions (β = − 1.00, SE = 0.22, CI(95%) = [− 1.44, − 0.56], t(203) =  − 4.50, p < 0.001) were negative predictors of sense of self-identification (η2c = 0.50, η2m = 0.08). Pairwise comparisons showed that participants in the Asynchronous condition had a higher sense of self-identification score than participants in the Synchronous condition (M_asynch_ = − 0.687, SE = 0.242, M_synch_ = 0.402, SE = 0.239, β = 1.0887, SE = 0.230, p = 0.0001), and the No-Body condition (M_nob_ = − 0.600, SE = 0.240, β = 1.0022, SE = 0.223, p = 0.0001). Our model also indicated that the Asynchronous (β = − 1.00, SE = 0.18, CI(95%) = [− 1.34, − 0.66], z = − 5.69, p < 0.001) and No-Body conditions (β = − 1.12, SE = 0.17, CI(95%) = [− 1.45, -0.80], z = − 6.75, p < 0.001) were negative predictors of sense of agency (η2c = 0.67, η2m = 0.20). Post hoc pairwise comparisons indicated that participants in the Asynchronous condition had a lower sense of agency than participants in the Asynchronous (M_asynch_ = 1.075, SE = 0. 227, M_synch_ = 2.049, SE = 0.224, β = − 0.973, SE = 0.173, p < 0.0001) and in the No-Body condition (M_nob_ = 0.953, SE = 0.223, β = − 1.095, SE = 0.163, p < 0.0001). Similarly, being in the Asynchronous condition (β = − 0.65, SE = 0.19, CI(95%) = [− 1.02, − 0.28], t = − 3.49, p < 0.001) and No-Body condition (β = − 0.44, SE = 0.18, CI(95%) = [− 0.79, − 0.09], t = − 2.46, p < 0.014) were negative predictors of sense of self-location (η2c = 0.67, η2m = 0.15). The pairwise comparison confirmed this result and indicated a lower sense of self-location for Asynchronous compared to Synchronous conditions (M_aynch_ = 0.831, SE = 0.257, M_synch_ = 1.481, SE = 0.255, β = 0.650, SE = 0.187, p = 0.0015) as well as a lower score in the No-Body compared to the Synchronous condition (M_nob_ = 1.039, SE = 0.255, β = 0.442, SE = 0.179, p = 0.0276) (Fig. 3A–C).

Analysis of the control questions revealed that being in the Asynchronous condition was a negative predictor of the feeling of having no body (β = − 0.29, SE = 0.13, CI(95%) = [− 0.55, − 0.03], t = − 2.18, p = 0.029) while being in the No-Body condition was a positive predictor (β = 0.26, SE = 0.12, CI(95%) = [0.02, 0.50], t = 2.14, p = 0.032) compared to the Synchronous condition (η2c = 0.88, η2m = 0.02). Pairwise comparisons showed a trend toward a higher score in the Asynchronous compared to the Synchronous condition (M_asynch_ = − 0.798, SE = 0.336, M_synch_ = − 0.510, SE = 0.335, β = 0.288, SE = 0.132, p = 0.0583) and the No-Body condition (M_nob_ = − 0.247, SE = 0.334, β = − 0.263, SE = 0.123, p = 0.0583), and also indicated a lower score in the Asynchronous compared to the No-Body condition (β = − 0.551, SE = 0.131, p = 0.0001). For the Two-bodies question, our model indicated that being in the Asynchronous condition was a positive predictor (β = 0.56, SE = 0.23, CI(95%) = [0.11, 1.01], z = − 2.45, p = 0.014; η2c = 0.58, η2m = 0.03). Pairwise comparisons confirmed this result and revealed a higher score for the Asynchronous compared to the Synchronous condition (M_aynch_ = 0.149, SE = 0.281, M_synch_ = − 0.716, SE = 0.278, β = -0.566, SE = 0.222, p = 0.0211) (Fig. 3D, E) and the No-Body condition (M_nob_ = -0.821, SE = 0.279, β = − 0.672, SE = 0.223, p = 0.0079).

### Sense of presence

**Figure 4:**
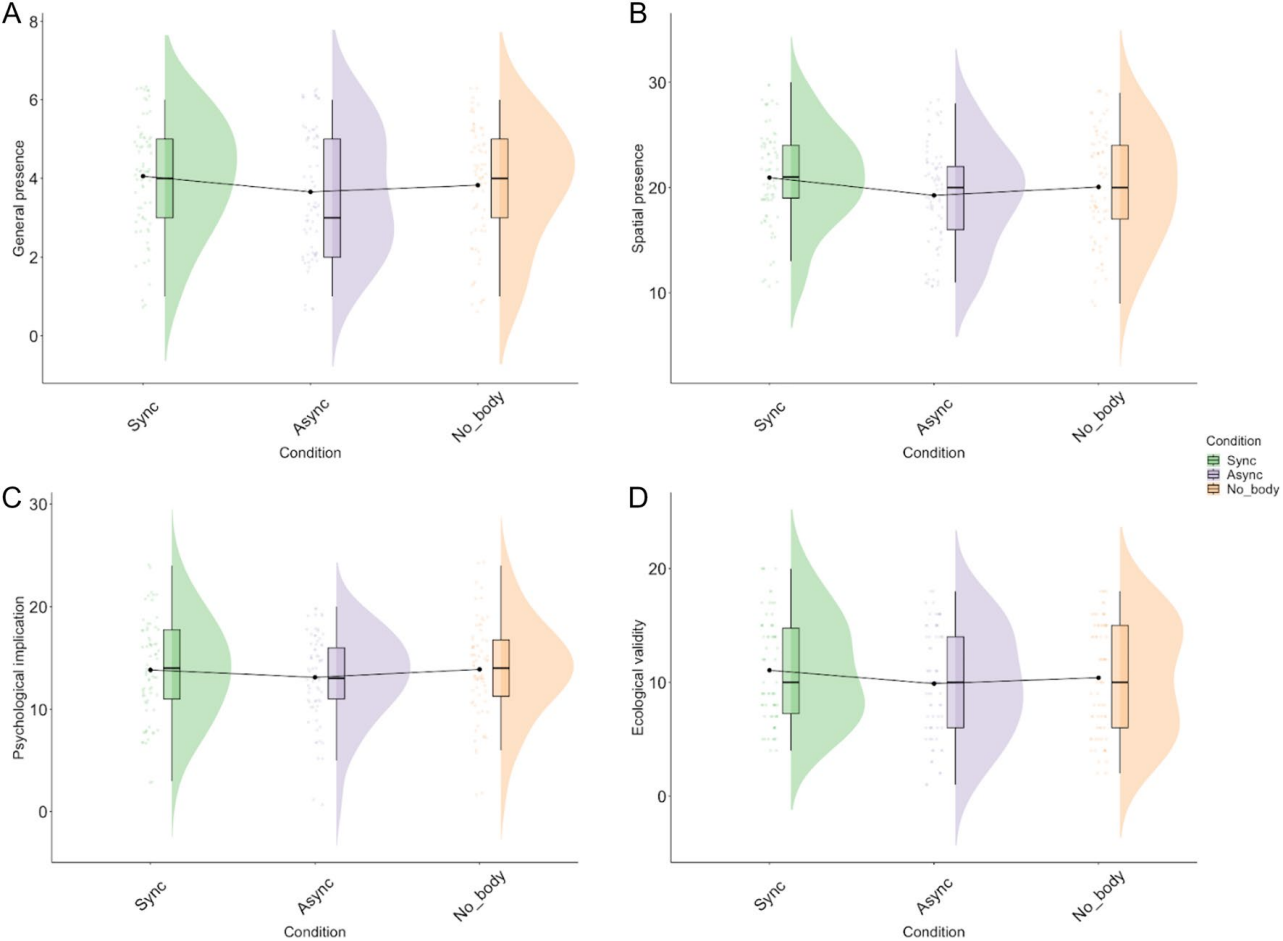
Results of the Igroup presence questionnaire.

Next, to test for differences in the sense of presence, we analyzed each subscore of the IPQ independently while controlling for the nausea subscore and navigation duration. The mixed model analysis revealed that the Asynchronous condition (β = − 0.51, SE = 0.12, CI(95%) = [− 0.75, − 0.27], t = − 3.54, p < 0.001) and the No-Body condition (β = − 0.27, SE = 0.12, CI(95%) = [− 0.50, − 0.04], t = − 2.27, p < 0.001) were significant negative predictors of the general sense of presence. (η2c = 0.80, η2m = 0.07). Pairwise comparisons indicated a lower sense of general presence in the Asynchronous compared to the Synchronous condition (M_synch_ = 4.17, SE = 0.241, M_asynch_ = 3.66, SE = 0.242, β = 0.509, SE = 0.124, p = 0.0001), and in the No-Body compared to the Synchronous condition (M_nob_ = 3.90, SE = 0.241, β = 0.267, SE = 0.118, p = 0.0469). Moreover, pairwise comparisons also indicated a trend toward a lower sense of general presence in the Asynchronous compared to the No-Body condition (β = -0.242, SE = 0.125, p = 0.0525). For the Spatial Presence subscore, our model revealed that being in the Asynchronous condition was a negative predictor of spatial presence (β = − 1.16, SE = 0.42, CI(95%) = [− 2.00, − 0.30], t = − 2.75, p = 0.006; η2c = 0.75, η2m = 0.06). Pairwise comparisons indicated that the sense of spatial presence was lower in Asynchronous than the Synchronous condition (M_synch_ = 20.9, SE = 0.726, M_asynch_ = 19.7, SE = 0.730, β = 1.164, SE = 0.424, p = 0.0181). Our model indicated similar results for ecological validity (β = − 1.24, SE = 0.40, CI(95%) = [− 2.03, − 0.44], t(203) =  − 3.06, p = 0.003; η2c = 0.77, η2m = 0.04). Pairwise comparisons indicated a lower sense of ecological validity in the Asynchronous compared to the Synchronous condition (M_synch_ = 11.02, SE = 0.732, M_asynch_ = 9.79, SE = 0.736, β = 1.235, SE = 0.404, p = 0.0078). Lastly, the Psychological Implication subscore did not indicate any significant differences between conditions (p > 0.05) (Fig. 4).

### Memory performances

To analyze memory performance, we fitted a model with Condition and Delay as a fixed effect and Subject as a random effect. For this model, in addition to the SSQ Nausea and Navigation Duration, we added the inter-session duration as a covariate, as it may vary substantially between participants.

#### Free recall

**Figure 5:**
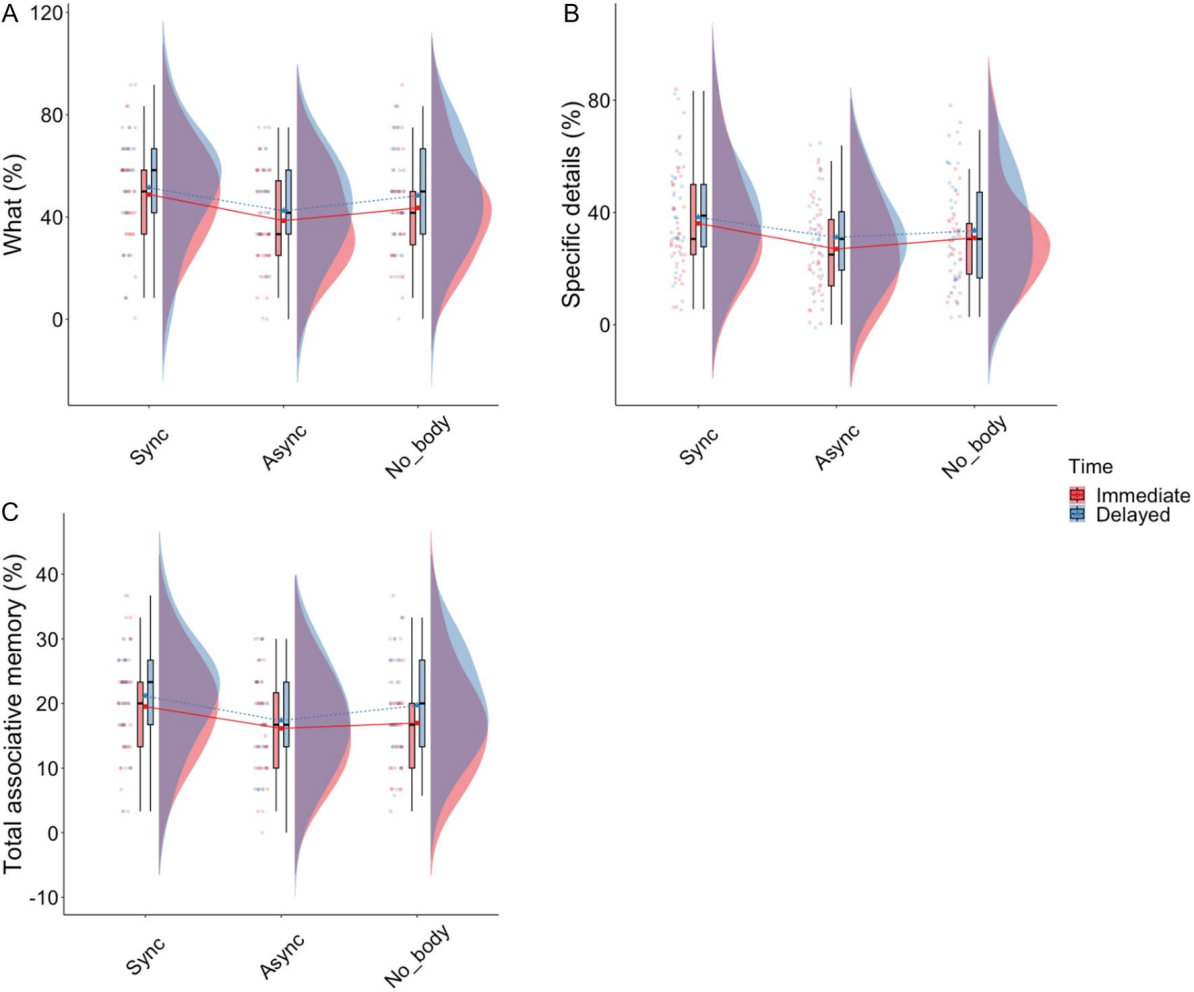
Results of the What, specific details and total association score of the EM free recall.

Our model indicated that being in the Asynchronous condition was a negative predictor of the number of events (What) recalled (β = − 9.99, SE = 4.07, CI5(95%) = [− 18.03, − 1.96], t(199) =  − 2.45, p = 0.015; η2c = 0.30; η2m = 0.07). Pairwise comparisons indicated a lower score in the Asynchronous compared to the Synchronous condition (M_asynch_ = 40.5, SE = 2.63, M_synch_ = 49.9, SE = 2.60, β = 9.4, SE = 2.93, p = 0.0047). There was no effect of the delay or interaction (Fig. 5A).

Regarding Egocentric Spatial and Temporal Situation memory, our model did not indicate any significant predictor. However, being in the Asynchronous condition was also a negative predictor of specific details (β = − 9.59, SE = 3.44, CI5(95%) = [− 16.32, − 2.85], t = − 2.79, p = 0.005; η2c = 0.39, η2m = 0.06). The pairwise comparison indicated that, on average, participants recalled fewer specific details in the Asynchronous compared to the Synchronous condition (M_asynch_ = 27.5, SE = 2.48, M_synch_ = 36.1, SE = 2.45, β = 8.57, SE = 2.47, p = 0.0016). The model did not indicate any other main effect or interaction (Fig. 5B).

Analysis of the mean total associative memory score revealed that being in the Asynchronous condition was a significant negative predictor of the total associative memory score. (β = − 3.49, SE = 1.66, CI5(95%) = [− 6.76, − 0.23], t(198) =  − 2.11, p = 0.036; η2c = 0.39, η2m = 0.06). Pairwise comparisons indicated a lower association score in the Asynchronous compared to the Synchronous condition (M_asynch_ = 16.6, SE = 1.008, M_synch_ = 20.3, SE = 0.995, β = 3.71, SE = 1.19, p = 0.0063). No other result was significant, and no effect of delay or interaction was found (Fig. 5C).

#### Recognition

**Figure 6:**
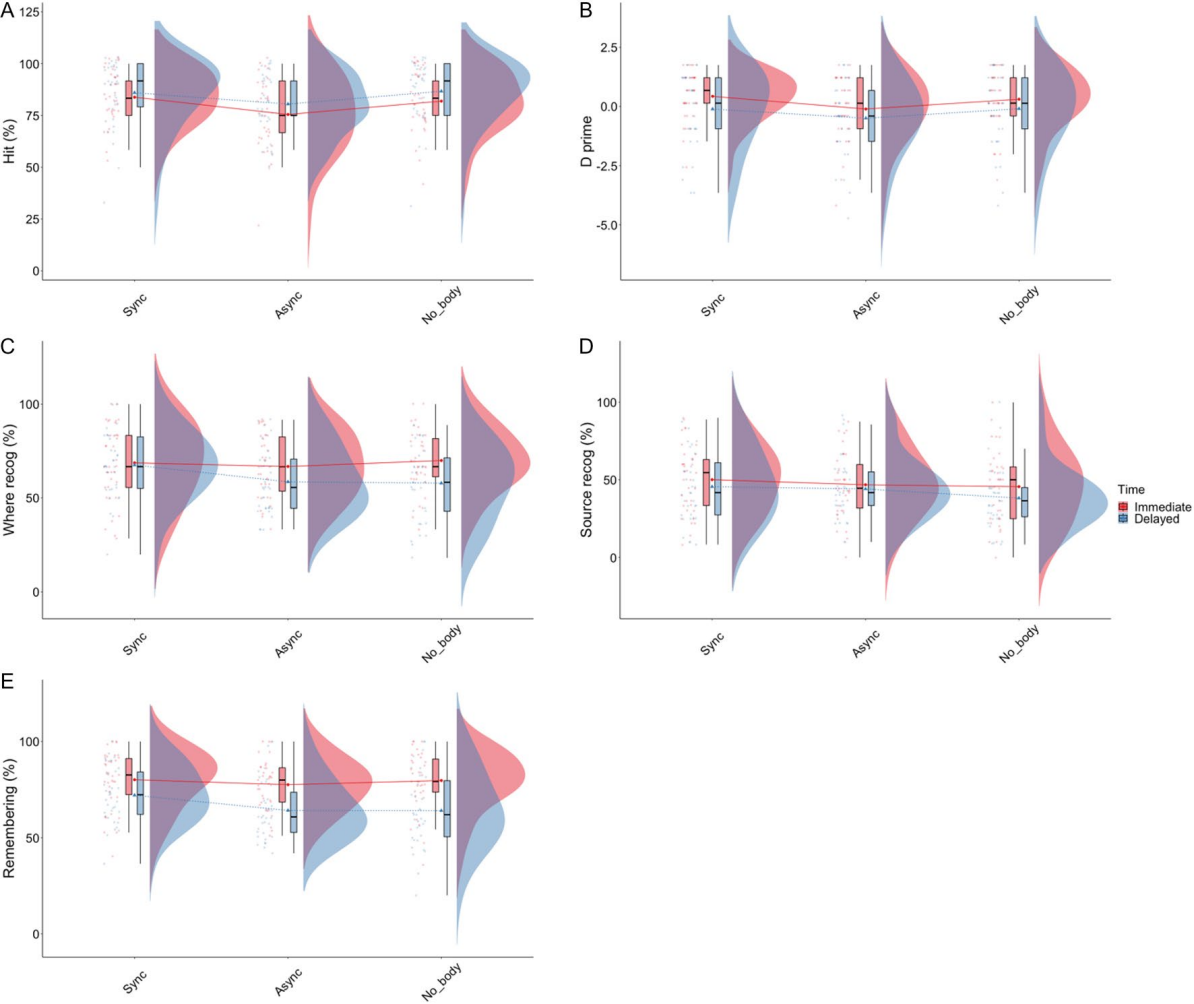
Results of the recognition tests.

Regarding the hit score, being in the Asynchronous condition was a negative predictor (β = − 6.94, SE = 2.95, CI(95%) = [− 12.71, − 1.16], t = − 2.35, p = 0.019; η2c = 0.42; η2m = 0.07). Pairwise comparisons further showed a lower hit score in the Asynchronous compared to the Synchronous condition (M_synch_ = 85.8, SE = 2.16, M_asynch_ = 79.5, SE = 2.19, β = 6.274, SE = 2.12, p = 0.0094), and a lower hit score in the Asynchronous compared to the No-Body condition (M_nob_ = 85.2, SE = 2.17, β = − 5.662, SE = 2.14, p = 0.0163) (Fig. 6A). Regarding false alarms, our model indicated that recall after a 10-day delay was a positive predictor of the false alarm rate (β = 4.85, SE = 0.94, CI(95%) = [3.01, 6.69], t = − 2.35, p < 0.001; η2c = 0.59; η2m = 0.18). Post hoc comparisons confirmed this result and indicated a lower False Alarm percentage in the Immediate compared to the Delayed condition (M_imm_ = 1.83, SE = 0.929, M_del_ = − 6.91, SE = 0.928, β = − 5.08, SE = 0.543, p < 0.0001). To assess overall performance in the recognition task, we calculated a d prime score based on the hit and false alarm scores. Our model indicated that being in the Asynchronous condition (β = − 0.41, SE = 0.22, CI(95%) = [− 0.84, 0.02], t = − 1.89, p = 0.059) or Delayed recall (β = − 0.44, SE = 0.21, CI(95%) = [− 0.85, − 0.03], t = − 2.08, p = 0.037) was a negative predictor of the d’ score (η2c = 0.63; η2m = 0.08). Post hoc comparisons indicated a trend toward a lower d’ score in the Asynchronous compared to the Synchronous condition (M_asynch_ = − 0.126, SE = 0.215, M_synch_ = 0.247, SE = 0.214, β = 0.3722, SE = 0.157, p = 0.0544), as well as a trend toward a lower score in the Asynchronous compared to the No-Body condition (M_nob_ = 0.193, SE = 0.214, β = − 0.3188, SE = 0.159, p = 0.0897). Moreover, the d’ score was higher in the Immediate, compared to the Delayed condition (M_imm_ = 0.30, SE = 0.204, M_del_ = -0.09, SE = 0.204, β = − 0.39, SE = 0.122, p = 0.0014) (Fig. 6B). Our model did not reveal any effect of delay or interaction between delay and condition.

Regarding contextual information, our model indicated that being in the No-Body condition was a negative predictor of egocentric spatial recall at 10 days delay (β = − 10.91, SE = 5.34, CI(95%) = [− 21.44, − 0.37], t(199) =  − 2.04, p = 0.043) (η2c = 0.31; η2m = 0.09). Post hoc comparisons indicated a lower score in the No-Body compared to the Synchronous condition (M_nob_ = 57.5, SE = 3.11, M_synch_ = 67.1, SE = 3.10, β = 9.631, SE = 3.79, p = 0.0361) as well as a trend toward a lower score in the Asynchronous compared to the Synchronous condition (M_asynch_ = 59.3, SE = 3.13, β = 7.805, SE = 3.84, p = 0.0873) at 10 days delay only (Fig. 6C). Analysis of temporal order and source recognition percentage did not reveal any significant main effect or interaction (All p > 0.05; See Supplementary Table S4). We did not find any other effect of delay or interaction between delay and condition. Lastly, we did not find any significant predictor regarding source condition (all p > 0.05) (Fig. 6D).

For sense of remembering, our model showed that recall at 10 days was a negative predictor of sense of remembering (β = − 8.08, SE = 2.65, CI(95%) = [− 13.30, − 2.86], t(199) =  − 3.05, p = 0.003). Moreover, our model revealed a significant interaction between Condition and Delay such that being in the No-Body condition (β = − 7.58, SE = 3.74, CI(95%) = [− 14.97, − 0.20], t(199) =  − 2.02, p = 0.044) was a negative predictor of sense of remembering at 10 days delay only (η2c = 0.64; η2m = 0.23). The post hoc test revealed that the sense of remembering was lower for the No-Body compared to the Synchronous condition (M_nob_ = 63.5, SE = 2.66, β = 8.137, M_synch_ = 71.6, SE = 2.66, SE = 2.66, p = 0.0078) and in the Asynchronous than in the Synchronous condition (M_asynch_ = 65.2, SE = 2.68, β = 6.420, SE = 2.71, p = 0.0381), but only after a 10-day delay (Fig. 6E).

Lastly, we analyzed mean subjective ratings of the events associated with a sense of remembering. Our model indicated that recall at 10 days was a negative predictor of memory Vividness, Confidence, Perspective, and Emotional intensity. Post hoc tests indicated a higher score for Immediate compared to Delayed recall for all measures (all p < 0.5, see Supplementary Table S4). No other effect of condition or interaction was found.

## Discussion

The role of bodily self-consciousness (BSC) in episodic memory (EM) has been the subject of increasing interest in recent years due to growing evidence suggesting that the body plays a crucial part in memory formation and retrieval^8,9^. In this study, we aimed to investigate further the impact of BSC on the incidental encoding of experienced specific events and its contribution to the objective, subjective, and associative components of EM by using immersion in a naturalistic virtual environment. By manipulating visuomotor synchronicity and body visibility to induce a body illusion over a personalized virtual avatar, participants navigated through a rich, multimodal naturalistic virtual environment in which a series of ecological, close-to-daily-life events were arranged. Our results revealed that synchronous visuo-motor stimulation positively enhances both objective and subjective components of EM compared to the Asynchronous condition and, to a lesser extent, the No-Body condition. In this discussion, we will first explore how our manipulations affected BSC in the three conditions, then we will focus on the comparison between Synchronous and Asynchronous conditions, followed by a discussion of the No-Body condition.

### BSC manipulation check and sense of presence

The results of our body-illusion questionnaire conform to our first hypothesis as the participants reported a lower sense of self-identification, a lower sense of agency, and a lower sense of being located within the avatar’s body in the Asynchronous and No-Body conditions compared to the Synchronous condition. These results confirm that our manipulation worked as expected and show that manipulating visuo-motor feedback on a virtual avatar effectively modulated all three components of BSC. Noteworthy, the self-identification score was positive only in the Synchronous condition, suggesting that it was the only condition where participants experienced a positive sense of identification with the virtual avatar. In contrast, sense of self-location and sense of agency scores were positive in all three conditions. For the No-Body condition, the positive agency score may be attributed to the presence of the virtual cellphone. The cellphone provided a minimal visual representation of the participants’ bodies in space, which may have helped them feel more embodied and experience a better sense of their actions. Additionally, the cellphone was synchronized with the participants’ movements, which may have helped them to feel more in control of their actions and to have a stronger sense of agency. For the Asynchronous condition, we expected sense of agency scores to follow self-identification as a sense of agency is known to depend on the comparison between the expected and actual multi-sensory consequences of motor commands, including their temporal dynamics^75^. Thus, as the visual feedback delay increases, the sense of agency should be diminished or abolished^76^. However, in our study, visuo-motor delay was kept constant, allowing participants to adjust to the altered temporal gap between their actions and consequences, thereby increasing their sense of agency over time^77^. Of note, although body parts and trunk movements were desynchronized, head movements and navigation controls remained synchronized with participants’ actions, which may have influenced participants while judging feelings of control over the virtual avatar. Nevertheless, large differences in questionnaire ratings between conditions may reflect various degrees of confidence or vividness of the illusion. Together, these results support the role of the multi-sensory integration of bodily signals for BSC and show that visuo-motor congruency may influence not only the sense of self-identification but also the sense of control and sense of self-location in space over an avatar seen from the 1PP.

Turning to the sense of presence, participants in the Synchronous condition had a higher feeling of general presence compared to the Asynchronous and No-Body conditions, as well as a higher sense of spatial presence and ecological validity compared to the Asynchronous but not the No-body condition. General presence refers to the subjective feeling of “being there” and measures the psychological immersion of the participants in the virtual environment. It has been argued that looking down at one’s body is strong evidence of being present in a given environment^78^. This result aligns with this claim and shows that multi-sensory congruency of body signals and body visibility are both important for sense of presence. Spatial presence, in turn, relates to the feeling of being physically present in a given environment^78^ and is tightly linked to body representation and the possibility of acting upon the immediate environment. Interacting with the environment requires monitoring the position of one’s body and effectors in relation to external objects^79^. Sense of body in space relies heavily on multi-sensory integration processes that blend spatial information about body position from various modalities^80^. Thus, one possible explanation for this result is that contrary to the No-Body condition, the discrepancy between visual and proprioceptive information in the Asynchronous condition may have prevented correct monitoring of the participant’s body in space due to ambiguous or conflicting body representations and disrupted sensorimotor coupling between the body, the self, and the world, leading to a lower sense of spatial presence and ecological validity. In support of this, a detailed inspection of our body illusion questionnaire revealed that the feeling of having no-body decreased from the No-Body to the Asynchronous condition. In contrast, participants in the Asynchronous condition had a higher feeling of having two bodies and a lower feeling of being in the avatar’s body. Another interpretation is that a lower sense of spatial presence and ecological validity results from higher cybersickness in the Asynchronous condition. However, in this case, we would have expected a similar pattern of results in the No-Body condition. Moreover, this result remains even when differences in cybersickness were controlled for.

Together, these results link the sense of bodily-self in space and a sense of presence. A strong sense of bodily self in space, as produced by synchronous visuomotor feedback, is associated with higher immersion, as well as a high psychological and physical sense of being “here” in the virtual environment. As sense of bodily-self decreases, the sense of psychological implication decreases while the sense of spatial presence and ecological validity remains. Lastly, a weak or conflicting bodily-self representation impairs both the psychological and physical sense of presence, as well as the participant’s feeling that the virtual environment is real. We will next discuss the relevance of these results for EM encoding.

### BSC and multifaceted episodic memory performance

The findings of the present EM tests offer partial support for our original hypotheses. Our results indicate that participants in the Asynchronous condition had a lower score on nearly all memory tests than those in the Synchronous condition. However, participants in the No-Body condition showed only slight deterioration in a few tests compared to those in the Synchronous condition. Consequently, our discussion will first focus on the differences between the Synchronous and Asynchronous conditions before analyzing the results from the No-Body condition separately.

#### The impact of Synchronous vs. Asynchronous condition

Concerning factual information, our results corroborate the findings of previous studies. We found that participants recalled fewer new events (i.e., specific scenes encountered during navigation) in the Asynchronous condition, regardless of the delay interval, and across both free recall and recognition tasks. More specifically, it was demonstrated through a lower proportion of event recall in the free recall task, a lower hit rate and a trend toward a lower d’ score in the recognition task, irrespective of the recall delay. This finding is consistent with previous studies showing that asynchronous visuo-tactile stimulation diminishes memory performance for factual information using words^52^, 3D objects^49,50^, and complex video scenes^53^, and extends this result to the incidental encoding of ecological events in naturalistic settings.

In addition to these results, our study reports several novel findings regarding the influence of bodily self-awareness on objective and subjective components of episodic memory.

First, we found that the free recall of events (What) was less often associated with multifaceted information (associative memory score), including contextual (Where, When) and specific details, in the Asynchronous condition compared to the Synchronous condition, showing for the first time that BSC may play a crucial role in the richness of EM traces (multiple features association). In fact, the Asynchronous condition significantly reduced the number of specific details recalled regardless of retention duration compared to the Synchronous condition. The present findings replicate those of Iriye and Ehrsson^53^, who found that asynchronous visuo-tactile stimulation during the encoding of an everyday-like scene decreased memory for central and peripheral details of the scene. Here, we extend these results to encoding ecological events in a naturalistic virtual environment in line with the view that the self-related bodily cues in the environment increase the event’s self-relevance of an experienced scene. Thus, the interaction between the BSC system and the memory system may help bind the various dimensions of experience into a well-integrated representation of the event in memory encoding, reinstatement, and long-term consolidation^81,82^. In support of this claim, Bréchet et al.^50^ found that seeing one's own body in a given environment retroactively strengthened memories of material encoded in this environment.

Concerning more specifically spatial information, our results from the recognition tests indicated that factual information tended to be less frequently linked to egocentric spatial information after a 10-day delay in the Asynchronous than the Synchronous condition. Remarkably, being in the Asynchronous condition altered the egocentric spatial reference frame^57^. According to the Scene Construction Hypothesis, reconstructing space is essential to EM and mental time travel^76^. Previous studies have shown that manipulating BSC modulates space perception, such as size estimation or depth perception. More recently, it has been demonstrated that changes in BSC are also associated with decreased spatial memory and spatial navigation performances. For example, Bergouignan and colleagues found that discrepancies between physical and seen body position during memory encoding may disturb binding processes in the hippocampus and impair EM for spatial, temporal, and emotional information^59^. It suggests that sense of bodily-self in space is essential for the EM of naturalistic events in context. In another study, Moon et al.^60^ immersed participants in a large virtual empty space where participants had to memorize the position of 3D objects while navigating freely. Notably, during the navigation, participants could see the body of a virtual mannequin in a supine position matching their physical body being stroked either synchronously or asynchronously. At retrieval, participants were again placed in the virtual environment and asked to navigate to the previous position of the object memorized. They demonstrated fewer distance errors and shorter path navigations when the participants saw the avatar associated with synchronous sensorimotor stimulation^60^.

Because of a ceiling effect, we failed to find significant differences between our conditions for egocentric spatial information in the free recall task. This means that when participants had to draw on internal resources to recall an event, it was quasi-systematically associated with correct egocentric spatial information. Future research could extend these investigations to different aspects of spatial information connected to EM, including visuospatial and allocentric measures (e.g., location of events on a map). Otherwise, there was no significant effect of conditions regarding specifically temporal information in the free recall and recognition tasks assessing the temporal order of events. These results depart from a recent study showing that BSC may modulate time perception^58^. However, this study investigates time duration estimation rather than sequential event memory, which was particularly difficult for our participants. Similar to spatial measurements, further studies could examine different types of temporal information to better understand the role of BSC in the precision of EM for temporal information^83^. Lastly, we found no difference between conditions regarding source memory. Our instructions directed participants' attention to the environment (which route) rather than their bodies (which condition). Consequently, it seems that none of our participants targeted visuo-motor changes as the primary goal of the experiment, leading to poor remembering of the condition. Future studies should explore additional measures to assess source information in greater detail.

Secondly, regarding the subjective dimensions of EM, we found that participants reported a higher sense of remembering (i.e., autonoetic recollection) in the Synchronous compared to the Asynchronous condition at delayed retrieval. Immediately after the first-person incidental encoding in VR, the sense of remembering was similar, whatever the BSC condition, but with a retention delay, there was a lower decrease in memory phenomenology in the Synchronous condition. This result supports previous VR studies demonstrating a link between body self-awareness and autonoetic consciousness for immediate and delayed recall^51,53^. For instance, Gauthier and colleagues used functional neuroimaging to measure changes in connectivity after encoding a virtual scene composed of a series of objects. They showed that post-encoding changes in connectivity strength between cerebral regions associated with BSC and the medial temporal lobe region correlated with the participant’s autonoetic recollection rating at one month delay only when the participant’s virtual body was visible during encoding^51^. Iriye and Ehrsson found that synchronous visuo-tactile stimulation over a virtual body seen from the first-person perspective enhanced the sense of reliving immediately after encoding and memory vividness, confidence, and emotional intensity at one week of delay^53^. However, their most recent study has not replicated these findings^84^. Regarding memory phenomenological scales, we did not reproduce all of Iriye and Ehrsson's results, as we only found a main effect of delay on the memory vividness, confidence, emotional intensity, reliving, and first-person perspective, but no effect of condition or interaction. One relevant explanation is that compared to Iriye and Ehrsson, our phenomenological evaluation of event memory retrieval was only proposed to participants with a remembering state attested by a score above 50 on the remembering scale. Consequently, all the participants who completed the phenomenological scales had a high level of remembering, which may have erased subtle differences in memory phenomenology in our participants.

Altogether, the present results reinforce our prior research on the effect of self-awareness on EM of naturalistic events using virtual reality^9,47,85^ and extend previous studies (see Table 1) revealing that BSC plays a crucial role in incidental encoding of naturalistic events supporting associative memory and autonoetic consciousness at retrieval, thus the objective and subjective properties of EM. These findings suggest, for the first time, that the perception of one's body in space may impact the level of detail in incidental memories of real-life events. The result of this study provides support to the claim that the integrated properties of self-perception may extend to the most basic form of bodily self-consciousness and improve the formation and retention of distinctive memory traces.

#### The special case of the No-Body condition

In addition to Synchronous and Asynchronous conditions, we also investigated a condition where the virtual body was removed from the participant’s first-person view. To date, no studies have directly compared Synchronous, Asynchronous, and No-Body conditions. Our results indicated that the No-Body condition is intermediate between the Synchronous and Asynchronous conditions. Specifically, we found that memory performance was better in the No-Body condition than in the Asynchronous condition regarding hit rates in the recognition task. Conversely, scores in the No-Body condition for egocentric spatial memory and sense of remembering were lower than in the Synchronous condition when the recall was delayed. No further differences were found regarding the other scores.

Before delving deeper into the possible reasons for this finding, it is essential first to discuss the nature of our No-Body condition. When participants are immersed in a virtual environment without a visible body, they must rely on proprioceptive and motor information to monitor the spatial position of their body. However, the lack of visual feedback for sensorimotor information may constitute a surprise event for the brain that may lead to a decrease in BSC. This is consistent with our data and findings showing that asynchronous visuo-tactile and visuomotor stimulation can reduce the sense of identification with one's own body^86^. Additionally, proprioceptive representations of the body in space are known to be less reliable than visual representation, which can further impair cognitive operations that rely on BSC. Only a few studies have investigated the impact of synchronous visuo-motor stimulation on memory performance compared to a No-Body condition^49–51^, but they have reported inconsistent results. Bréchet et al.^49,50^ found that occluding participants’ bodies while encoding objects in a virtual room decreased memory performance in a subsequent surprise recognition task. However, Gauthier et al.^51^ failed to replicate this result using a similar procedure. Moreover, as a subjective BSC measure was not assessed in these studies, it is impossible to ensure that changes in memory performances were specifically related to changes in the sense of bodily self.

Nevertheless, a significant difference between our study and the studies cited above is to be noted. As mentioned above, participants in each condition, including the No-Body condition, were equipped with a virtual cellphone placed in their right-hand during navigation. This procedure was chosen to motivate participants to use their virtual bodies and to ensure the comparability of our experimental conditions. Although the avatar’s body was occluded in the No-Body condition, the cellphone moved congruently and synchronously with the participant's physical hand. Thus, congruent spatial and temporal feedback from the cellphone may have been sufficient to help participants to build a more stable and accurate bodily-self representation in the No-Body compared to the Asynchronous condition, as shown by their higher sense of spatial presence. Indeed, it has been demonstrated that synchronous multi-sensory stimulation may elicit a sense of self-identification over an invisible body^66^. Similarly, Kondo et al.^87^ found that the mere perception of spatially congruent but disconnected hands and feet might suffice to create a sense of self-identification, sense of agency, and sense of self-location over the corresponding interpolated body. Thus, we suggest that minimal visuo-motor cues, as provided by the spatially congruent position of the cellphone, may have been sufficient for participants to form a weak but coherent representation of the body in space that helped them to construct an organized and integrated memory trace from encoding and in turn, helped them to form a coherent representation of the event in memory.

An alternative explanation is that the additional cognitive resources required to adapt to the desynchronization of visual and motor information in the Asynchronous condition may have specially altered event encoding. Consistent with this claim is that participants in the Asynchronous condition exhibited longer navigation duration, which is a consequence of higher cognitive load^40^. However, by including this parameter as a covariate in our analysis, we accounted for a portion of the differences in cognitive load between our conditions. Furthermore, the extent to which this explanation is valid is unclear. For example, adaptation time to a mismatch can be very rapid, with some studies reporting adaptation after only a few trials^88^ Moreover, it has been shown that visuomotor adaptation rate is robust to cognitive load^89^. Thus, it is unlikely that visuo-motor adaptation was disturbed by the experimental task. In the present study, participants in the Asynchronous condition performed nearly 80 movements during the induction phase, which is likely to have been sufficient for adaptation to occur. Finally, an explanation centered on executive functions cannot explain the lower performance in remembering and egocentric spatial memory in the No-Body condition. Therefore, while the present study does not completely rule out the possibility that cognitive demands played a role in the impairment in event encoding in the Asynchronous condition compared to the No-Body one, it is likely to have played a minor role. Nevertheless, further research is needed to determine the extent to which visuomotor adaptation can account for the effect of BSC on EM.

### New direction for a theoretical framework of the effects of BSC on EM

Our study unravels the links between sense of bodily-self in space and EM of naturalistic events by highlighting that BSC supports memory performance (i.e., number of events, richness of details, associative information, and discriminability), and preserves contextual information and sense of remembering over time. How can BSC account for these results?

Neuroimaging studies have identified a network of interconnected brain regions, including frontoparietal, temporoparietal, and subcortical areas associated with BSC, with different sub-networks supporting spatial and self-components of BSC^90^. These regions dynamically integrate available sensory signals from different modalities to build a coherent sense of being a self in a body and have been associated with a variety of functions including spatial navigation, EM and sense of self in space^91–93^. Interestingly, recent behavioral and neuroimaging studies suggest that the multi-sensory processes involved in BSC are integrated into the memory trace to support encoding and episodic remembering^66,94^. As such, it has been argued that the parietal areas support the integration of multimodal memory features within an egocentric framework into the kind of first-person-perspective representation that enables the subjective reexperiencing of past events^95^. For example, it was found that subjective reports of self-location correlated with the increased connectivity between posterior parietal and hippocampal activity. Similarly, Moon et al.^60^ found that first-person perspective navigation decreased entorhinal cortex activity and increased retrosplenial cortex activity, linked with spatial navigation and spatial memory performances. Moreover, Bergouignan et al.^59,96^ found that experimentally induced out-of-body experience reduced hippocampal activity during retrieval and the ability to remember events from a field perspective. It suggests that multi-sensory processes in the posterior parietal regions are conveyed to the hippocampus to build a coherent representation of the bodily-self, which may increase the richness of the memory trace, leading to better performance in our Synchronous condition compared to the other two. In parallel, interactions with subnetworks related to the sense of agency and sense of self-identification may signal the self-relevance of the event^9,49,84^, enhancing associative encoding for intra-event features and contextual information, increasing memory precision and preventing memory decay over time^50^. Finally, at retrieval, these processes are reinstated or reenacted, producing a rich and vivid recollection of the event centered around the bodily-self and imbued with a sense of agency, and autonoetic consciousness. This claim was supported by a recent neuroimaging study by Iriye et al.^84^. Using a similar paradigm as Iriye and Ehrsson^53^, they found increased similarity between encoding and retrieval activity for highly vivid memories in brain regions including the hippocampus and the bilateral angular gyrus, when events were encoded in the synchronous condition. The authors concluded that sense of bodily-self in space during encoding is likely a fundamental contextual memory cue based on spatial relationships that delineate oneself from the external world^84^. Continuing previous research^9^, we argue that the multifaceted self-reference effect via grounding personal everyday experiences through bodily self in space and narrative self is crucial to promote the formation of the self-memory system and long-lasting EM (i.e., episodic autobiographical memory).

### Limitations and future directions

The current study offers a unique perspective on the impact of bodily self-consciousness on EM, but there are some limitations that may have influenced the results and call for further investigation. Firstly, we cannot entirely rule out the possibility that our findings result partially from increased cognitive demand in the Asynchronous condition. Future research could therefore explore the relationship between multi-sensory processing, executive function, and EM, as this issue may not be restricted to visuo-motor stimulation. The implementation of control devices such as eye-tracking systems could also help to track differences in environmental exploration between conditions. Another solution would be to manipulate interoceptive rather than exteroceptive signals to modulate BSC^97,98^. Secondly, our study did not enable us to disentangle the specific role of each BSC component on EM. A thorough examination of the role played by self and spatial components of BSC would help the field to better understand the link between BSC and EM and its functions. Thirdly, we need to substantiate how these findings are transferable to real life and contribute to the long-lasting EM, also referred to as episodic autobiographical memory^2,6^. While EM can refer to any event that happened to us in the past and any contextual learning of new information, in contrast, episodic autobiographical memory concerns self-relevant specific experienced events^99^. Future research should examine the role of BSC in EM according to the self-relevance of experienced events. Overall, our study provides a foundation for further exploration of the role of bodily self-consciousness in naturalistic episodic memory, but additional research is needed to address these limitations and expand our understanding of this complex phenomenon.

## Conclusion

In conclusion, our study provides new insights into the relationship between bodily-self in space and memory of incidental encoding of specific events in a naturalistic environment. We found that manipulating the visuomotor feedback associated with BSC during encoding can modulate the subsequent EM performance, even at long retention delay, showing for the first time that BSC may play a role in feature binding and autonoetic consciousness associated with the memory of everyday events. Our findings are in accordance with previous studies showing the importance of multi-sensory integration for EM and suggest that the bodily-self representation plays a crucial role in this process. However, future work is needed to confirm our results as some issues persist regarding that underly EM alteration performance when BSC is degraded and discard alternative explanations. This research may have implications for the design of virtual environments as tools for learning and rehabilitation. By optimizing the feedback signals that promote BSC, it may be possible to optimize the encoding and retention of complex events^75^. Furthermore, our study may contribute to the understanding of memory deficits in bodily-self disorders such as depersonalization and schizophrenia, which are characterized by a disruption of the integration between body signals^100^. Future research could investigate whether similar effects on memory consolidation occur in these populations and whether virtual reality interventions could help to alleviate their cognitive impairments. Overall, this study highlights the importance of considering the multi-sensory processes that build the sense of bodily-self as an integral part of the encoding and retrieval processes in memory consolidation, and it opens up new avenues for investigating the neural and cognitive mechanisms underlying this phenomenon.

### Supplementary Information

Supplementary Tables.

## Data Availability

The data of this study are available on request to the corresponding authors.
