# Supplementary material for: The role of bodily self-consciousness in episodic memory of naturalistic events: an immersive virtual reality study
Source: Sci Rep. 2023 Oct 9;13:17013. doi: 10.1038/s41598-023-43823-2 (PMC10562507; doi:10.1038/s41598-023-43823-2)
Supplement: Supplementary file 1 — Supplementary Tables. [file 41598_2023_43823_MOESM1_ESM.docx]

| Scores | Synch | Asynch | No-body |
| --- | --- | --- | --- |
| Duration | 8.44 (0.273)  [7.90 – 8.97] | 9.23 (0.273)  [8.70 – 9.77] | 8.39 (0.273)  [7.86 – 8.93] |
| SSQ_total | 7.63 (1.06)  [5.54 – 9.71] | 8.58 (1.06)  [6.49 – 10.67] | 8.54 (1.06)  [6.45 –10.63] |
| SSQ_occulomotor | 4.51 (0.532)  [3.47 – 5.56] | 4.98 (0.532)  [3.94 – 6.02] | 4.96 (0.532)  [3.91 – 6.00] |
| SSQ_nausea | 3.22 (0.564)  [2.21 – 4.33] | 3.84 (0.564)  [2.73 – 4.94] | 3.80 (0.564)  [2.70 – 4.91] |

**The role of bodily self-consciousness in episodic memory of naturalistic events: An immersive virtual reality study**

Sylvain Penaud^1*^, Delphine Yeh^1^, Alexandre Gaston-Bellegarde^1^, Pascale Piolino^1*^

^1^ Université Paris Cité, Laboratoire Mémoire, Cerveau & Cognition, F-92100 Boulogne-Billancourt, France

**Supplementary materials**

**Table S1** – *Navigation duration and Simulator sickness questionnaire scores. The table present marginal estimated means, standard error and 95% confidence interval per condition for navigation duration and Simulator Sickness Questionnaire.*

| Scores | Synch | Asynch | No-body |
| --- | --- | --- | --- |
| Self-identification | 0.402 (0.239)  [-0.0763 – 0.880] | -0.687 (0.242)  [-1.1702 – -0.204] | -0.600 (0.240)  [-1.0794 – -0.122] |
| Agency | 2.049 (0.224)  [1.609 – 2.49] | 1.075 (0.227)  [0.631 – 1.52] | 0.953 (0.223)  [0.515 –1.39] |
| Self-location | 1.481 (0.255)  [0.981 – 1.98] | 0.831 (0.257)  [0.327 – 1.33] | 1.039 (0.255)  [0.539 – 1.54] |
| Nobody | -0.405 (0.327)  [-1.046 – 0.2364] | -0.741 (0.328)  [-1.384 – -0.0981] | -0.237 (0.327)  [-0.879 – 0.4046] |
| Two-bodies | -0.716 (0. 278)  [-1.26 – -0.170] | -0.149 (0. 281)  [-0.70 – 0.401] | -0.821 (0. 279)  [-1.37 – -0.274] |

**Table S2** – *Body-illusion questionnaire scores. The table present marginal estimated means, standard error and 95% confidence interval per condition for the body-illusion questionnaire.*

| **Scores** | **Synch** | **Asynch** | **No-body** |
| --- | --- | --- | --- |
| **General presence** | 4.17 (0.241)  [3.69 – 4.64] | 3.66 (0.242)  [3.18 – 4.13] | 3.90 (0.241)  [3.43 – 4.37] |
| **Spatial presence** | 20.9 (0.726)  [19.4 – 22.3] | 19.7 (0.730)  [18.3 – 21.1] | 20.4 (0.727)  [19.0 – 21.8] |
| **Ecological validity** | 13.8 (0.711)  [12.3 – 15.2] | 13.0 (0.716)  [11.5 – 14.4] | 14.1 (0.712)  [12.7 – 15.5] |
| **Psychological implication** | 11.02 (0.732)  [9.54 – 12.5] | 9.79 (0.736)  [8.30 – 11.3] | 10.53 (0.732)  [9.05 – 12.0] |

**Table S3** – *Igroup presence questionnaire scores. The table present marginal estimated means, standard error and 95% confidence interval per condition for the Igroup Presence Questionnaire*

| **Scores** | **Delay** | **Synch** | **Asynch** | **No-Body** | **Mean** |
| --- | --- | --- | --- | --- | --- |
| *Recognition* | | | | | |
| **Hits** | Immediate | 84.1 (2.60)  [79.1 – 89.2] | 77.2 (2.62)  [72.1 – 82.4] | 82.8 (2.60)  [77.7 – 87.9] | 81.4 (1.98)  [77.5 – 85.3] |
|  | Delayed | 87.4 (2.60)  [82.3 – 92.5] | 81.8 (2.62)  [76.6 – 86.9] | 87.5 (2.60)  [82.4 – 92.6] | 85.6 (1.98)  [81.7 – 89.4] |
| **FA** | Immediate | 1.83 (1.07)  [-0.27 – 3.93] | 1.93 (1.08)  [-0.18 – 4.05] | 1.80 (1.07)  [-0.30 – 3.90] | 1.86 (0.924)  [0.0445 – 3.67] |
|  | Delayed | 6.68 (1.07)  [4.58 – 8.78] | 81.8 (2.62)  [76.6 – 86.9] | 87.5 (2.60)  [82.4 – 92.6] | 6.95 (0.924)  [5.1394 – 8.76] |
| **D’** | Immediate | 0.4666 (0.238)  [-0.000708 – 0.934] | 0.0562 (0.240)  [-0.414211 – 0.527] | 0.3757 (0.239)  [-0.092140 – 0.844] | 0.30 (0.204)  [-0.10 – 0.699] |
|  | Delayed | 0.0266 (0.238)  [-0.440670 – 0.494] | -0.3074 (0.240)  [-0.777806 – 0.163] | 0.0107 (0.239)  [-0.457171 – 0.479] | -0.09 (0.924)  [-0.49 – 0.310] |
| **Where (%)** | Immediate | 68.3 (3.10)  [62.2 – 74.5] | 67.5 (3.13)  [61.3 – 73.7] | 69.6 (3.11)  [63.4 – 75.7] | 68.5 (2.19)  [64.1 – 72.9] |
|  | Delayed | 67.1 (3.10)  [61.0 – 73.3] | 59.3 (3.13)  [53.1 – 65.5] | 57.5 (3.11)  [51.4 – 63.6] | 61.3 (2.19)  [56.9 – 65.7] |
| **When (%)** | Immediate | 23.1 (2.88)  [17.5 – 28.8] | 19.9 (2.89)  [14.2 – 25.5] | 24.7 (2.88)  [19.1 – 30.4] | 22.6 (1.66)  [19.3 – 25.8] |
|  | Delayed | 22.9 (2.88)  [17.3 – 28.6] | 19.2 (2.89)  [13.5 – 24.8] | 20.9 (2.88)  [15.2 – 26.5] | 21.0 (1.66)  [17.7 – 24.2] |
| **Source (%)** | Immediate | 50.0 (3.78)  [42.6 – 57.4] | 46.4 (3.79)  [39.0 – 53.9] | 45.1 (3.78)  [37.6 – 52.5] | 47.2 (2.17)  [42.9 – 51.4] |
|  | Delayed | 44.7 (3.78)  [37.3 – 52.1] | 42.4 (3.79)  [35.0 – 49.8] | 36.9 (36.9)  [29.5 – 44.3] | 41.3 (2.17)  [37.1 – 45.6] |
| **Remembering** | Immediate | 79.7 (2.66)  [74.4 – 85.0] | 78.6 (2.68)  [73.3 – 83.99] | 79.1 (2.66)  [73.9 – 84.4] | 79.1 (2.16)  [74.8 – 83.5] |
|  | Delayed | 71.6 (2.66)  [66.3 – 76.9] | 65.2 (2.68)  [59.9 – 70.5] | 63.5 (2.66)  [58.2 – 68.8] | 66.8 (2.16)  [62.4 – 71.16] |
| *Phenomenology* | | | | | |
| **Reliving** | Immediate | 83.4 (2.31)  [78.8 – 87.9] | 80.8 (2.32)  [76.2 – 85.4] | 80.3 (2.31)  [75.8 – 84.8] | 81.5 (2.01)  [77.6 – 85.4] |
|  | Delayed | 71.6 (2.66)  [66.3 – 76.9] | 65.2 (2.68)  [59.9 – 70.5] | 63.5 (2.66)  [58.2 – 68.8] | 73.4 (2.01)  [69.5 – 77.4] |
| **Perspective** | Immediate | 81.8 (3.28)  [75.4 – 88.2] | 77.7 (3.29)  [71.3 – 84.2] | 81.9 (3.28)  [75.4 – 84.8] | 80.5 (2.94)  [74.7 – 86.2] |
|  | Delayed | 78.9 (3.28)  [72.5 – 85.3] | 79.4 (3.29)  [72.9 – 85.8] | 82.1 (3.28)  [75.6 – 88.5] | 80.1 (2.94)  [74.4 – 85.9] |
| **Vividness** | Immediate | 79.5 (2.48)  [74.6 – 84.5] | 76.6 (2.50)  [71.6 – 81.6] | 77.6 (2.48)  [72.6 – 82.5] | 77.9 (2.15)  [73.6 – 82.3] |
|  | Delayed | 70.4 (2.48)  [65.5 – 75.4] | 70.6 (2.50)  [65.6 – 75.6] | 72.0 (2.48)  [67.0 – 76.9] | 71.0 (2.15)  [66.6 – 75.39] |
| **Fidelity** | Immediate | 74.1 (2.70)  [68.8 – 79.4] | 74.3 (2.72)  [69.0 – 79.6] | 74.4 (2.70)  [69.1 – 79.7] | 74.3 (2.42)  [69.5 – 79.0] |
|  | Delayed | 67.2 (2.70)  [61.9 – 61.9] | 67.3 (2.72)  [61.9 – 72.6] | 68.2 (2.70)  [62.9 – 73.5] | 67.6 (2.42)  [62.8 – 72.3] |
| **Emotional intensity** | Immediate | 50.8 (3.67)  [43.4 – 58.2] | 49.6 (3.68)  [42.2 – 57.0] | 50.9 (3.67)  [43.5 – 58.3] | 50.4 (3.4)  [43.5 – 57.3] |
|  | Delayed | 48.8 (3.67)  [41.4 – 56.2] | 47.8 (3.68)  [40.4 – 55.2] | 44.9 (3.67)  [37.5 – 52.3] | 47.2 (3.4)  [40.3 – 54.1] |
| **Memory ownership** | Immediate | 61.8 (4.01)  [53.9 – 69.6] | 61.8 (4.03)  [53.9 – 69.7] | 63.5 (4.02)  [55.6 – 71.4] | 62.4 (3.74)  [55.0 – 69.7] |
|  | Delayed | 58.2 (4.01)  [50.4 – 66.1] | 57.3 (4.03)  [49.4 – 65.2] | 58.3 (4.02)  [50.4 – 66.1] | 57.9 (3.74)  [50.6 – 65.3] |
| *Free recall* | | | | | |
| **What (%)** | Immediate | 48.6 (3.28)  [42.1 – 55.1] | 38.6 (3.31)  [32.1 – 45.1] | 43.7 (3.29)  [37.3 – 50.2] | 43.7 (2.31)  [39.0 – 48.3] |
|  | Delayed | 51.2 (3.28)  [44.7 – 57.7] | 42.4 (3.31)  [35.9 – 49.0] | 48.5 (3.29)  [42.0 – 55.0] | 47.4 (2.31)  [42.8 – 52.0] |
| **Where (%)** | Immediate | 95.6 (1.70)  [92.3 – 98.9] | 96.1 (1.71)  [92.8 – 99.5] | 93.5 (1.70)  [90.4 – 97.1] | 95.2 (1.08)  [93.1 – 97.3] |
|  | Delayed | 92.2 (1.70)  [88.9 – 95.6] | 95.1 (1.71)  [91.7 – 98.4] | 93.5 (1.70)  [90.2 – 96.8] | 93.6 (1.08)  [91.5 – 95.7] |
| **When (%)** | Immediate | 48.2 (5.27)  [37.8 – 58.5] | 49.0 (5.32)  [38.6 – 59.5] | 44.8 (5.27)  [34.4 – 55.1] | 47.3 (3.63)  [40.2 – 54.5] |
|  | Delayed | 44.7 (5.27)  [34.4 – 55.1] | 48.7 (5.32)  [38.3 – 59.2] | 44.3 (5.28)  [33.9 – 54.6] | 45.9 (3.63)  [38.8 – 53.0] |
| **Details** | Immediate | 34.9 (2.98)  [29.1 – 40.7] | 25.3 (3.00)  [19.4 – 31.2] | 29.7 (2.98)  [23.9 – 35.5] | 30.0 (3.63)  [25.6 – 34.4] |
|  | Delayed | 37.2 (2.98)  [31.4 – 43.1] | 29.7 (3.00)  [23.8 – 35.6] | 33.5 (2.98)  [27.6 – 39.3] | 33.5 (2.24)  [29.1 – 37.8] |
| **Mean association** | Immediate | 19.5 (1.29)  [17.0 – 22.0] | 16.0 (1.30)  [13.4 – 18.6] | 17.2 (1.31)  [14.6 – 19.8] | 17.6 (0.873)  [15.8 – 19.3] |
|  | Delayed | 21.1 (1.29)  [18.6 – 23.7] | 17.2 (1.30)  [14.6 – 19.8] | 19.8 (1.29)  [17.3 – 22.4] | 19.4 (0.870)  [17.6 – 21.1] |

**Table S4** – *Recognition and free recall scores. The table present marginal estimated means, standard error and 95% confidence interval per condition for free recall and recognition test both at immediate and 10 day delay.*
